# Supplementary material for: The Microbiota and Cytokines Correlation between the Jejunum and Colon in Altay Sheep
Source: Animals (Basel). 2022 Jun 17;12(12):1564. doi: 10.3390/ani12121564 (PMC9219508; doi:10.3390/ani12121564)
Supplement: Supplementary file 1 [file animals-12-01564-s001.zip › animals-1738918-supplementary.pdf]

Table S1: P values of correlations between microbial populations and cytokines in the jejunum.

| | IL_2 | IL_6 | IL_10 | IL_12 | TNF_$\alpha$ |
|---|---|---|---|---|---|
| *g__Paeniclostridium* | 0.544 | 0.266 | 0.544 | 0.329 | 0.623 |
| *g__Pseudomonas* | 0.623 | 0.623 | 0.397 | 0.872 | 0.704 |
| *g__unclassified_f__Peptostreptococcaceae* | 0.156 | 0.111 | 0.787 | 0.329 | 0.623 |
| *g__Candidatus_Saccharimonas* | 0.872 | 0.872 | 0.623 | 0.397 | 0.208 |
| *g__norank_c__WCHB1-41* | 0.397 | 0.623 | 0.208 | 0.872 | 0.957 |
| *g__norank_f__vadinBE97* | 0.208 | 0.208 | 0.623 | 0.208 | 0.266 |
| *g__Succinivibrio* | 0.957 | 0.425 | 0.700 | 0.173 | 0.257 |
| *g__Ruminococcaceae_UCG-014* | 0.623 | 0.623 | 0.872 | 0.623 | 0.468 |
| *g__norank_f__Rhodospirillaceae* | 0.787 | 0.468 | 0.544 | 0.787 | 0.957 |
| *g__Aeriscardovia* | 0.544 | 0.468 | 0.787 | 0.329 | 0.208 |
| *g__unclassified_f__Coriobacteriaceae* | 0.397 | 0.623 | 0.208 | 0.872 | 0.957 |
| *g__Succiniclasticum* | 0.658 | 0.461 | 0.577 | 0.321 | 0.425 |
| *g__Mogibacterium* | 0.208 | 0.397 | 0.397 | 0.872 | 0.787 |
| *g__Clostridium_sensu_stricto_1* | 0.397 | 0.208 | 0.872 | 0.397 | 0.787 |
| *g__norank_o__Gastranaerophilales* | 0.623 | 0.623 | 0.872 | 0.623 | 0.468 |
| *g__norank_f__Bifidobacteriaceae* | 0.957 | 0.827 | 0.008 ** | 0.784 | 0.577 |
| *g__Senegalimassilia* | 0.329 | 0.266 | 0.957 | 0.156 | 0.266 |
| *g__unclassified_f__Bifidobacteriaceae* | 0.518 | 0.439 | 0.686 | 0.295 | 0.295 |
| *g__Olsenella* | 0.872 | 0.623 | 0.872 | 0.208 | 0.266 |
| *g__Family_XIII_AD3011_group* | 0.787 | 0.468 | 0.957 | 0.329 | 0.397 |
| *g__Ruminococcus_2* | 0.787 | 0.957 | 0.019 * | 0.957 | 0.704 |
| *g__Saccharofermentans* | 0.872 | 0.872 | 0.072 | 0.623 | 0.329 |
| *g__Ureaplasma* | 0.468 | 0.329 | 0.957 | 0.704 | 0.872 |
| *g__norank_f__Ruminococcaceae* | 0.397 | 0.623 | 0.208 | 0.872 | 0.957 |
| *g__[Eubacterium]_coprostanoligenes_group* | 0.872 | 0.623 | 0.872 | 0.397 | 0.329 |
| *g__Solobacterium* | 0.518 | 0.439 | 0.686 | 0.295 | 0.295 |
| *g__Erysipelotrichaceae_UCG-009* | 0.787 | 0.468 | 0.544 | 0.787 | 0.957 |
| *g__Turicibacter* | 0.329 | 0.266 | 0.787 | 0.329 | 0.544 |
| *g__unclassified_k__norank* | 0.957 | 0.704 | 0.544 | 0.329 | 0.208 |
| *g__Cellulosilyticum* | 0.468 | 0.156 | 0.704 | 0.266 | 0.623 |

Correlations between microbial populations and cytokines detected in the jejunum of Altay sheep ($n = 12$). * Statistical significance is indicated by * $p < 0.05$, ** $p < 0.01$.

Table S2: R values of correlations between microbial populations and cytokines in the jejunum.

| | IL_2 | IL_6 | IL_10 | IL_12 | TNF_a |
|---|---|---|---|---|---|
| *g__Paeniclostridium* | −0.314 | −0.543 | −0.314 | −0.486 | −0.257 |
| *g__Pseudomonas* | 0.257 | 0.257 | −0.429 | 0.086 | −0.200 |
| *g__unclassified_f__Peptostreptococcaceae* | −0.657 | −0.714 | −0.143 | −0.486 | −0.257 |
| *g__Candidatus_Saccharimonas* | 0.086 | 0.086 | 0.257 | 0.429 | 0.600 |
| *g__norank_c__WCHB1-41* | −0.429 | −0.257 | 0.600 | −0.086 | −0.029 |
| *g__norank_f__vadinBE97* | −0.600 | −0.600 | 0.257 | −0.600 | −0.543 |
| *g__Succinivibrio* | −0.029 | −0.406 | −0.203 | −0.638 | −0.551 |
| *g__Ruminococcaceae_UCG-014* | −0.257 | −0.257 | −0.086 | −0.257 | −0.371 |
| *g__norank_f__Rhodospirillaceae* | −0.143 | −0.371 | −0.314 | −0.143 | 0.029 |
| *g__Aeriscardovia* | −0.314 | −0.371 | −0.143 | −0.486 | −0.600 |
| *g__unclassified_f__Coriobacteriaceae* | −0.429 | −0.257 | 0.600 | −0.086 | −0.029 |
| *g__Succiniclasticum* | −0.232 | −0.377 | 0.290 | −0.493 | −0.406 |
| *g__Mogibacterium* | 0.600 | 0.429 | 0.429 | 0.086 | 0.143 |
| *g__Clostridium_sensu_stricto_1* | −0.429 | −0.600 | −0.086 | −0.429 | −0.143 |
| *g__norank_o__Gastranaerophilales* | −0.257 | −0.257 | −0.086 | −0.257 | −0.371 |
| *g__norank_f__Bifidobacteriaceae* | −0.029 | 0.116 | 0.928 | 0.145 | 0.290 |
| *g__Senegalimassilia* | 0.486 | 0.543 | −0.029 | 0.657 | 0.543 |
| *g__unclassified_f__Bifidobacteriaceae* | −0.334 | −0.395 | 0.213 | −0.516 | −0.516 |
| *g__Olsenella* | 0.086 | −0.257 | −0.086 | −0.600 | −0.543 |
| *g__Family_XIII_AD3011_group* | −0.143 | −0.371 | 0.029 | −0.486 | −0.429 |
| *g__Ruminococcus_2* | −0.143 | −0.029 | 0.886 | 0.029 | 0.200 |
| *g__Saccharofermentans* | 0.086 | 0.086 | 0.771 | 0.257 | 0.486 |
| *g__Ureaplasma* | 0.371 | 0.486 | 0.029 | 0.200 | −0.086 |
| *g__norank_f__Ruminococcaceae* | −0.429 | −0.257 | 0.600 | −0.086 | −0.029 |
| *g__[Eubacterium]_coprostanoligenes_group* | −0.086 | −0.257 | −0.086 | −0.429 | −0.486 |
| *g__Solobacterium* | −0.334 | −0.395 | 0.213 | −0.516 | −0.516 |
| *g__Erysipelotrichaceae_UCG-009* | −0.143 | −0.371 | −0.314 | −0.143 | 0.029 |
| *g__Turicibacter* | −0.486 | −0.543 | −0.143 | −0.486 | −0.314 |
| *g__unclassified_k__norank* | 0.029 | −0.200 | −0.314 | −0.486 | −0.600 |
| *g__Cellulosilyticum* | −0.371 | −0.657 | −0.200 | −0.543 | −0.257 |

Pearson's correlation analysis of microbial populations and cytokines in in the colon ($n$ = 12).

Table S3: P values of correlations between microbial populations and cytokines in the colon.

| | IL_2 | IL_6 | IL_10 | IL_12 | TNF_$\alpha$ |
|---|---|---|---|---|---|
| *g__Ruminococcaceae_UCG-010* | 0.266 | 0.397 | 0.072 | 0.468 | 0.397 |
| *g__Prevotella_1* | 0.111 | 0.468 | 0.208 | 0.329 | 0.787 |
| *g__Fibrobacter* | 0.544 | 0.468 | 0.329 | 0.623 | 0.042 * |
| *g__Christensenellaceae_R-7_group* | 0.468 | 0.872 | 0.704 | 0.111 | 0.872 |
| *g__Ruminococcaceae_UCG-005* | 0.872 | 0.704 | 0.872 | 0.042 * | 0.957 |
| *g__Rikenellaceae_RC9_gut_group* | 0.787 | 0.704 | 0.111 | 0.623 | 0.957 |
| *g__norank_f__Bacteroidales_BS11_gut_group* | 0.872 | 0.329 | 0.623 | 0.111 | 0.468 |
| *g__unclassified_f__Lachnospiraceae* | 0.704 | 0.872 | 0.329 | 0.208 | 0.397 |
| *g__Treponema_2* | 0.957 | 0.872 | 0.208 | 0.468 | 0.072 |
| *g__Phocaeicola* | 0.872 | 0.872 | 0.872 | 0.957 | 0.208 |
| *g__Bacteroides* | 0.623 | 0.957 | 0.544 | 0.156 | 0.704 |
| *g__Alistipes* | 0.787 | 0.872 | 0.872 | 0.329 | 0.072 |
| *g__Akkermansia* | 0.872 | 0.329 | 0.623 | 0.111 | 0.468 |
| *g__norank_f__Bacteroidales_UCG-001* | 0.544 | 0.468 | 0.329 | 0.623 | 0.042 * |
| *g__norank_f__Clostridiales_vadinBB60_group* | 0.019 * | 0.397 | 0.156 | 0.872 | 0.872 |
| *g__[Eubacterium]_coprostanoligenes_group* | 0.397 | 0.329 | 0.544 | 0.468 | 0.111 |
| *g__Phascolarctobacterium* | 0.872 | 0.544 | 0.957 | 0.266 | 0.019 * |
| *g__Ruminococcaceae_NK4A214_group* | 0.787 | 0.872 | 0.623 | 0.019 * | 0.872 |
| *g__Prevotellaceae_UCG-001* | 0.329 | 0.329 | 0.072 | 0.266 | 0.957 |
| *g__Elusimicrobium* | 0.787 | 0.872 | 0.623 | 0.019 * | 0.872 |
| *g__unclassified_f__Ruminococcaceae* | 0.266 | 0.872 | 0.397 | 0.329 | 0.872 |
| *g__unclassified_p__Bacteroidetes* | 0.872 | 0.329 | 0.266 | 0.397 | 0.544 |
| *g__norank_f__Marinilabiaceae* | 0.305 | 0.034 * | 0.268 | 0.468 | 0.468 |
| *g__Barnesiella* | 0.704 | 0.704 | 0.397 | 0.468 | 0.111 |
| *g__Ruminococcaceae_UCG-002* | 0.156 | 0.872 | 0.872 | 0.957 | 0.623 |
| *g__norank_o__Gastranaerophilales* | 0.208 | 0.266 | 0.042 * | 0.872 | 0.544 |
| *g__Prevotellaceae_UCG-004* | 0.389 | 0.913 | 0.036 * | 0.354 | 0.913 |
| *g__Prevotellaceae_UCG-003* | 0.872 | 0.208 | 0.623 | 0.787 | 0.005 ** |
| *g__Ruminococcaceae_UCG-014* | 0.111 | 0.042 * | 0.468 | 0.544 | 0.111 |
| *g__Ruminococcaceae_UCG-013* | 0.072 | 0.005 ** | 0.397 | 0.957 | 0.208 |

Correlations between microbial populations and cytokines detected in the colon of Altay sheep ($n$ = 12). * Statistical significance is indicated by * $p < 0.05$, ** $p < 0.01$.

Table S4: R values of correlations between microbial populations and cytokines in the colon.

| | IL_2 | IL_6 | IL_10 | IL_12 | TNF_α |
|---|---|---|---|---|---|
| *g__Ruminococcaceae_UCG-010* | −0.543 | 0.429 | 0.771 | −0.371 | −0.429 |
| *g__Prevotella_1* | 0.714 | −0.371 | −0.600 | 0.486 | 0.143 |
| *g__Fibrobacter* | −0.314 | 0.371 | −0.486 | −0.257 | −0.829 |
| *g__Christensenellaceae_R-7_group* | −0.371 | 0.086 | 0.200 | −0.714 | −0.086 |
| *g__Ruminococcaceae_UCG-005* | −0.086 | −0.200 | 0.086 | −0.829 | −0.029 |
| *g__Rikenellaceae_RC9_gut_group* | −0.143 | 0.200 | 0.714 | 0.257 | 0.029 |
| *g__norank_f__Bacteroidales_BS11_gut_group* | 0.086 | −0.486 | 0.257 | −0.714 | 0.371 |
| *g__unclassified_f__Lachnospiraceae* | 0.200 | 0.086 | −0.486 | −0.600 | −0.429 |
| *g__Treponema_2* | 0.029 | −0.086 | 0.600 | 0.371 | 0.771 |
| *g__Phocaeicola* | 0.086 | −0.086 | −0.086 | 0.029 | −0.600 |
| *g__Bacteroides* | 0.257 | −0.029 | −0.314 | 0.657 | −0.200 |
| *g__Alistipes* | 0.143 | 0.086 | 0.086 | −0.486 | −0.771 |
| *g__Akkermansia* | −0.086 | 0.486 | −0.257 | 0.714 | −0.371 |
| *g__norank_f__Bacteroidales_UCG-001* | 0.314 | −0.371 | 0.486 | 0.257 | 0.829 |
| *g__norank_f__Clostridiales_vadinBB60_group* | −0.886 | 0.429 | 0.657 | 0.086 | −0.086 |
| *g__[Eubacterium]_coprostanoligenes_group* | −0.429 | 0.486 | −0.314 | −0.371 | −0.714 |
| *g__Phascolarctobacterium* | 0.086 | 0.314 | 0.029 | −0.543 | −0.886 |
| *g__Ruminococcaceae_NK4A214_group* | 0.143 | −0.086 | 0.257 | −0.886 | 0.086 |
| *g__Prevotellaceae_UCG-001* | −0.486 | 0.486 | 0.771 | −0.543 | −0.029 |
| *g__Elusimicrobium* | −0.143 | 0.086 | −0.257 | 0.886 | −0.086 |
| *g__unclassified_f__Ruminococcaceae* | −0.543 | −0.086 | 0.429 | −0.486 | 0.086 |
| *g__unclassified_p__Bacteroidetes* | −0.086 | 0.486 | 0.543 | 0.429 | 0.314 |
| *g__norank_f__Marinilabiaceae* | −0.507 | 0.845 | 0.541 | −0.372 | −0.372 |
| *g__Barnesiella* | 0.200 | −0.200 | −0.429 | −0.371 | −0.714 |
| *g__Ruminococcaceae_UCG-002* | −0.657 | −0.086 | −0.086 | 0.029 | −0.257 |
| *g__norank_o__Gastranaerophilales* | −0.600 | 0.543 | 0.829 | −0.086 | −0.314 |
| *g__Prevotellaceae_UCG-004* | −0.435 | 0.058 | 0.841 | −0.464 | 0.058 |
| *g__Prevotellaceae_UCG-003* | 0.086 | −0.600 | 0.257 | 0.143 | 0.943 |
| *g__Ruminococcaceae_UCG-014* | −0.714 | 0.829 | 0.371 | −0.314 | −0.714 |
| *g__Ruminococcaceae_UCG-013* | −0.771 | 0.943 | 0.429 | −0.029 | −0.600 |

Pearson's correlation analysis of microbial populations and cytokines in in the colon ($n$ = 12).
